# Characterization of the interatrial septum by high-field cardiac MRI: a comparison with multi-slice computed tomography

**DOI:** 10.1186/s43044-020-00109-6

**Published:** 2020-11-12

**Authors:** Abdalla Elagha, Yaseen Othman, Reham Darweesh, Ghada Awadein, Assem Hashad

**Affiliations:** 1 Cardiovascular Department (Kasr-Alainy Hospital), Cairo University, 1 Saraya St., Third floor, Manial, Cairo, Egypt; 2 Barts and the London School of Medicine and Dentistry, London, England

**Keywords:** Interatrial septum (IAS), Cardiac MRI (CMR), High-field CMR, 3 tesla, Multi-slice computed tomography (MSCT)

## Abstract

**Background:**

Assessment of the interatrial septum (IAS) has become an attractive area of interest for a variety of important interventional procedures. Newer imaging modalities like multi-slice computed tomography (MSCT) and cardiac MRI (CMR) can provide higher resolution and wider field of view than echocardiography. Moreover, high-field (3-Tesla) CMR can even enhance spatial and temporal resolution.

The characteristics of the interatrial septum were retrospectively studied in 371 consecutive subjects (201 men, 31–73 years old) in whom MSCT was performed primarily for non-invasive evaluation of the coronary arteries. All subjects underwent both MSCT and MRI scans within 0–30 day’s interval. A 3D volume covering the whole heart was acquired across the heart with and without contrast enhancement. Also, patients underwent cardiac MSCT examinations using 64-row MSCT scanners.

**Results:**

The mean scan time of MSCT was 10.4 ± 2.8 s and 9.7 ± 2.9 min for CMR. The mean length of IAS by CMR and CT was 39.65 ± 4.6 mm and 39.28 ± 4.7 mm, respectively. The mean maximal thickness of IAS by CMR and CT was 3.1 ± 0.97 mm and 3.15 ± 0.95 mm, respectively. The mean thickness of fossa ovalis by CMR and CT was 1.04 ± 0.36 mm and 1.04 ± 0.44 mm, respectively. The mean length of fossa ovalis by CMR and CT was 12.8 ± 3.7 mm and 12.8 ± 3.5 mm, respectively. Finally, the mean angle of IAS by CMR and CT was identical (155 ± 9.2°). Measurements of various morphological features of IAS showed no statistically significant difference between CMR and CT, with an excellent correlation and close relationship regarding IAS length, maximal IAS thickness, fossa ovalis thickness, fossa ovalis length, and IAS angle (*r* = 0.98, 0.98, 0.95, 0.96, and 0.92, respectively).

**Conclusion:**

Whole-heart 3D acquisition at 3-T MRI using a free-breathing technique provides a valuable non-invasive imaging tool for excellent assessment of the interatrial septum—as compared to MSCT—that may have significant clinical implication for diagnostic purposes and therapeutic interventional procedures, as it may facilitate planning, improve outcome, and shorten its duration.

## Background

Assessment of the interatrial septum (IAS) has become an important requirement for the diagnosis of a variety of congenital and pathological diseases, as well as being considered an essential and attractive spot for a variety of important interventional procedures. These procedures demand precise and detailed characterization of the septum in order to facilitate the selection of procedure-specific devices, e.g., transseptal puncture [1–3] (a crucial step before performing radiofrequency ablation of the left atrium, mitral valvuloplasty, and repair) and closure of atrial septal defects [4], which subsequently increased the need for accurate pre-procedural assessment of atrial septum anatomy, morphology, and its spatial relationships. Appropriate knowledge of the IAS anatomy before these procedures would facilitate, improve outcome, and shorten their duration [5].

Echocardiography is an established tool for cardiac imaging, but it has narrow fields of view, has a limited acoustic window, and is considered semi-invasive when using the transesophageal (TEE) approach, which may result in an accidental esophageal tear, rupture, or cardio-respiratory arrest [6].

Multi-slice computed tomography (MSCT) has an excellent spatial resolution and wide field of view, but unfortunately carries the risk of using ionizing radiation and the use of potentially nephrotoxic iodinated contrast agent.

On the other hand, cardiac magnetic resonance imaging (CMR) has become a powerful clinical tool in the assessment of the cardiovascular system as it allows for non-invasive, multi-planar imaging with high intrinsic contrast, and most importantly, avoiding the hazards of MSCT [7–9].

The advent of 3-Tesla (3-T) MRI scanners made a significant impact in the cardiac MRI field, as imaging at higher field strength has the potentials of improved imaging outcome by enhancing signal-to-noise ratio (SNR) and contrast-to-noise ratio (CNR), which enhances spatial and temporal resolution [10, 11]. Available MR techniques mostly utilize breath-holding 2D techniques with relatively low spatial resolution.

The purpose of this study is to demonstrate the feasibility of using a free-breathing 3-dimensional (3D) technique at 3-T MRI for the assessment of interatrial septum. The specific objectives of this study were to set up normal reference values for anatomical and morphological features of the interatrial septum using 3-T CMR and to compare it with MSCT measurements.

## Methods

### Study population

The characteristics of the interatrial septum were retrospectively studied in 371 consecutive subjects (201 men, 31–73 years old) in whom MSCT was performed primarily for non-invasive evaluation of the coronary arteries. Informed written consent was obtained from each individual regarding participation in the CMR examination. Subjects over 18 years old with known or suspected atherosclerotic disease were included in our study. Exclusion criteria included subjects with general contraindication to MRI or MSCT scanning, iodinated or gadolinium contrast use, and beta-blocker intolerance. Electrocardiograms were interpreted as a normal sinus rhythm for all participants.

### Study design

All subjects underwent both MSCT and MRI scans within 0–30-day interval, with the majority (292 subjects) having both scans on the same day. Neither urgent medications nor adverse clinical events were recorded between two scans. Patients not already on beta-blocking drugs received 2.5–10 mg of bisoprolol for heart rates over 65 beats/min at rest, 1 h before MSCT imaging.

### MR imaging protocol

Previously described scanning protocols in the literature were used in this study with minor modifications [12, 13]. All patients were imaged on two 3-T MRI scanners (Philips Medical Systems, Best, NL) using a multi-channel cardiac phased-array receiver coil.

### Cardiac gating

Vector electrocardiographic (VCG) was utilized for gating in all scans, which allow reliable R-wave triggering and overcome enhanced ECG changes at 3 T (due to the amplified magneto-hydrodynamic effect at 3 T) [14].

### Respiratory motion suppression

As CMR examination was acquired with no breath-hold, all cases were done with free breathing. For compensation of respiratory motion, 2D selective RF pulse with 12 revolutions in *k*-space and a beam radius of 15 mm was used [15]. A real-time navigator beam was positioned at the dome of the right hemidiaphragm with an acceptance window of 5 mm.

### Localizers

For localization, a multi-slice gradient-echo technique acquiring three orthogonal stacks with transversal, sagittal, and coronal slice orientation was used. Based on these scout images, the navigator was positioned at the dome of the right hemidiaphragm and the position of the imaged volume for whole-heart imaging. The following parameters were used: TR = 11 ms; TE = 2.4 ms; *α* = 20**°**.

### Trigger delay and coil sensitivity profile

The optimal trigger delay (TD) is estimated visually at the most quiescent period in the diastolic resting period (at about 75% of the cardiac cycle) during a retrospectively gated steady-state free precession (SSFP) four-chamber view cine image.

### 3D whole-heart MRA scan

After the scout and preparatory scans, 3D whole-heart and corrected 3D segmented *k*-space gradient echo was acquired using the visually identified TD. Volumetric shimming was used for 3D acquisitions. Also, parallel imaging (sensitivity encoding; SENSE acceleration factor of 2) was utilized.

A 3D volume covering the whole heart (50 partitions of 2-mm thickness interpolated to 100 partitions of 1-mm thickness during reconstruction) was acquired across the heart with an acquired voxel size of 1 × 1 × 2 mm^3^. A detailed description of the acquisition parameters is provided in Table 1. For all sequences, repetition rates and flip angles were set to operate within the manufacturer set SAR limits.

**Table 1:** Acquisition parameters for the contrast-enhanced whole-heart MRI

***Parameter***	***Value***
Repetition time TR (ms)	4.4
Echo-time TE (ms)	1.5
Flip angle (°)	20
Inversion recovery (ms)	220
Pixel bandwidth (Hz)	299.5
Acquisition window (ms)	150
TFE factor	34
Resolution (mm^3^)	1 × 1 × 2
SENCE factor	2

Whole-heart imaging was performed with and without contrast enhancement. For non-contrast MRI, a cardiac-triggered, segmented, adiabatic T2-prepared technique was applied. For the contrast-enhanced imaging (for coronary MR angiography and viability test), the T2 preparation was replaced by an inversion recovery (IR) preparation. Regarding contrast infusion, each subject received up to a dose of 0.2 mmol/kg of gadolinium-based contrast agent (gadoterate meglumine, Guerbet, France)

### CT imaging protocol

Patients underwent a cardiac MSCT examination using a 64-row MSCT scanner with a 0.33-s gantry rotation time (SOMATOM Sensation 64, Siemens, Germany) and images acquired with retrospective ECG gating. All CT scans were performed with patients in supine position and feet toward the gantry. Starting at the level of the carina, craniocaudal scanning (coverage length 80–110 mm) was performed. Technical parameters used were the following: collimation 32 × 2 × 0.6 mm, slice thickness 0.75 mm, increment 0.4 mm, gantry rotation time 0.33 s, pitch 0.2, 800 mAs, and 120 kVp. For the MSCT angiogram, 70–100 mL of nonionic iodinated contrast (Isovue-370, Bracco Diagnostics Inc, Italy) was injected followed by saline solution. Scanning was performed during breath-holding.

Images were reconstructed at 70–80% phases of the cardiac cycle which corresponds to most quiescent interval of the diastolic rest period. Retrospective electrocardiographic gating was performed to eliminate cardiac motion artifacts. Data acquisition was completed in about 10–12 s.

### Interatrial septum analysis

Analysis of both CMR and CT images was performed using a dedicated workstation and commercially available imaging analysis software (Viewforum, Philips). The CT and CMR measurements were performed using the same measuring tool on that software. The images of all patients were randomly reviewed by consensus. For a more comprehensive assessment of the interatrial septum, axial CT images were rendered into a four-chamber view, where interatrial septum and fossa ovalis lengths were measured. The IAS thickness was measured anterior and posterior to the fossa ovalis, and the maximal thickness was recorded.

**Fig. 1:**
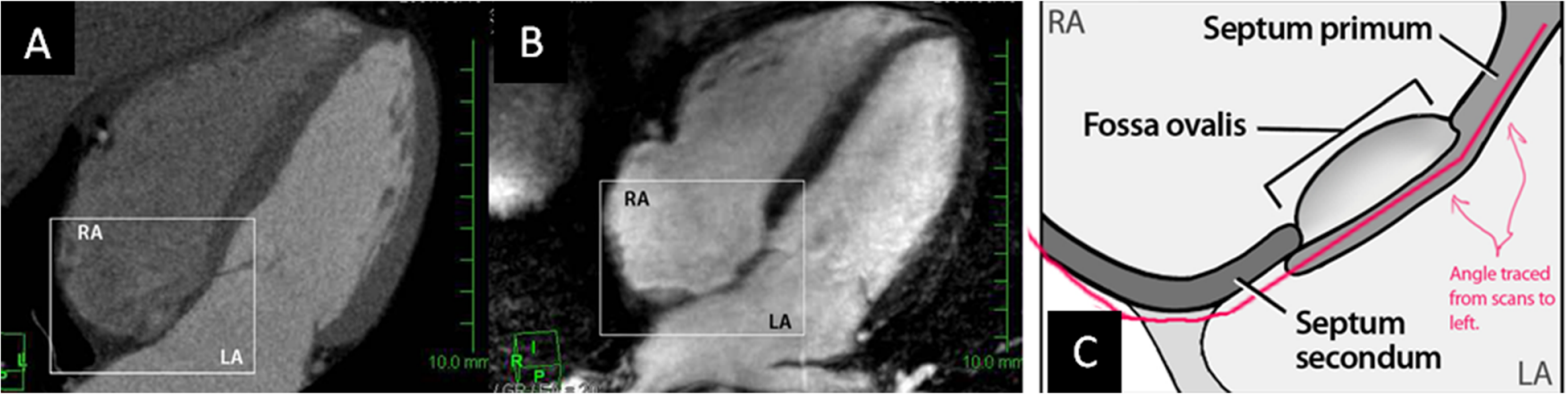
Interatrial septum, as shown by **a** MSCT, **b** MRI, and **c** illustrated sketch diagram. The latter shows detailed anatomical and morphological features of the IAS. LA, left atrium; RA, right atrium

In the four-chamber view, the interatrial septum appears as a slightly curved line from the junction between the left ventricle and right atrium, coursing posterior and to the right, making an obtuse angle at the meeting point of the septum primum and the limbus of fossa ovalis (Fig. 1). The IAS angle was measured at mid-diastole.

### Statistical analysis

Linear regression analysis was performed to determine the relationship and correlation between interatrial septum measurements obtained by CMR and MSCT. Medcalc software was used to generate a scatter plot. Continuous parameters were reported as mean ± standard deviation and compared using two-tailed paired *t* tests. Categoric variables were reported as frequencies and percentages. A *p* value ≤ 0.05 was considered statistically significant.

## Results

All patients underwent both CMR and MSCT without complications. Patients’ characteristics are shown in Table 2.

**Table 2:** Patients’ characteristics of all subjects who underwent both CMR and MSCT scans

***Patient characteristics***	
Age (years), mean ± SD	55 ± 9
Gender, M/F	201/170
Weight (kg), mean ± SD	88.4 ± 17
Pervious MI, *n* (%)	22 (6%)
Hypertension, *n* (%)	230 (62%)
Diabetes mellitus, *n* (%)	78 (21%)
Dyslipidemia, *n* (%)	182 (49%)
Smoking, *n* (%)	159 (43%)

According to the scope of this study, which is to compare both imaging modalities in IAS assessment, we are not reporting here the results of the coronary assessment. No patients had to be excluded because of suboptimal image quality.

The images from total 742 scans (CMR and MSCT) of all 371 subjects were analyzed in axial and 4-chamber views and randomly reviewed by consensus of two experts (blinded to other results) in cardiac CT and CMR. The mean scan time of MSCT with contrast was 10.4 ± 2.8 s. For CMR, the mean value of navigator efficiency was 49%, and the mean acquisition time of the whole-heart scan was 9.7 ± 2.9 min.

Accidentally, a patent foramen ovale was visualized in 6 patients using MSCT; however, CMR confirmed 5 of them. No atrial septal defects, atrial septal aneurysms, or lipomatous hypertrophy of the interatrial septum were encountered.

The mean length of the interatrial septum by CMR and CT was 39.65 ± 4.6 mm and 39.28 ± 4.7 mm, respectively. The mean maximal thickness of the interatrial septum by CMR and CT was 3.1 ± 0.97 mm and 3.15 ± 0.95 mm, respectively. The mean thickness of fossa ovalis by CMR and CT was 1.04 ± 0.36 mm and 1.04 ± 0.44 mm, respectively. The mean length of fossa ovalis by CMR and CT was 12.8 ± 3.7 mm and 12.8 ± 3.5 mm, respectively. Finally, the mean angle of IAS (located between primum and secundum septae) by CMR and CT was identical (155 ± 9.2°).

**Fig. 2:**
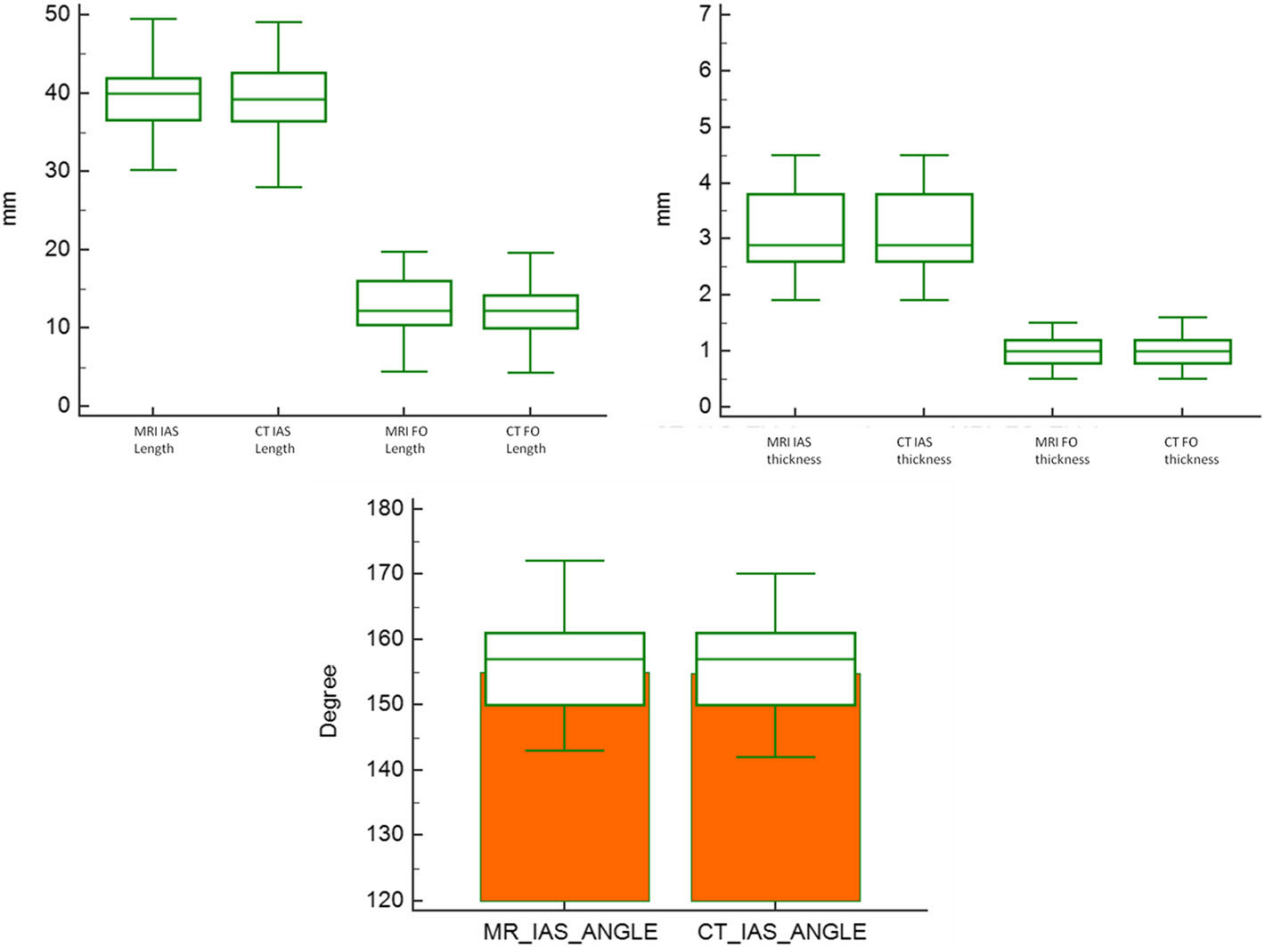
Comparison graphs of both techniques. It demonstrates the close relationship between various measurements of the interatrial septum (IAS) obtained using CMR and MSCT IAS length, maximal IAS thickness, fossa ovalis thickness, fossa ovalis length, and IAS angle. FO, fossa ovalis; IAS, interatrial septum

Measurements of various morphological features of IAS are shown in Table 3, which illustrated that there was no statistically significant difference (*p* = not significant) between CMR and CT regarding all measurements. Figure 2 shows comparable findings in both groups.

**Table 3:** Measurements of various interatrial septum characteristics by 3-T MRI and MSCT. All measurements are shown as mean ± SD

	MRI	MSCT	***p*** value
IAS length (mm)	39.65 **± 4.6**	39.28 **± 4.7**	NS
IAS thickness (mm)	3.1 **± 0.97**	3.15 **± 0.95**	NS
FO thickness (mm)	1.04 **± 0.36**	1.04 **± 0.44**	NS
FO length (mm)	12.8 **± 3.7**	12.8 **± 3.5**	NS
IAS angulations (degrees)	155 **± 9.2**	155 **± 9.2**	NS

At the same time, there was an excellent correlation and close relationship between measurements obtained by both CMR and MSCT, e.g., IAS length, maximal IAS thickness, fossa ovalis thickness, fossa ovalis length, and IAS angle (*r* = 0.98, 0.98, 0.95, 0.96, and 0.92, respectively).

**Fig. 3:**
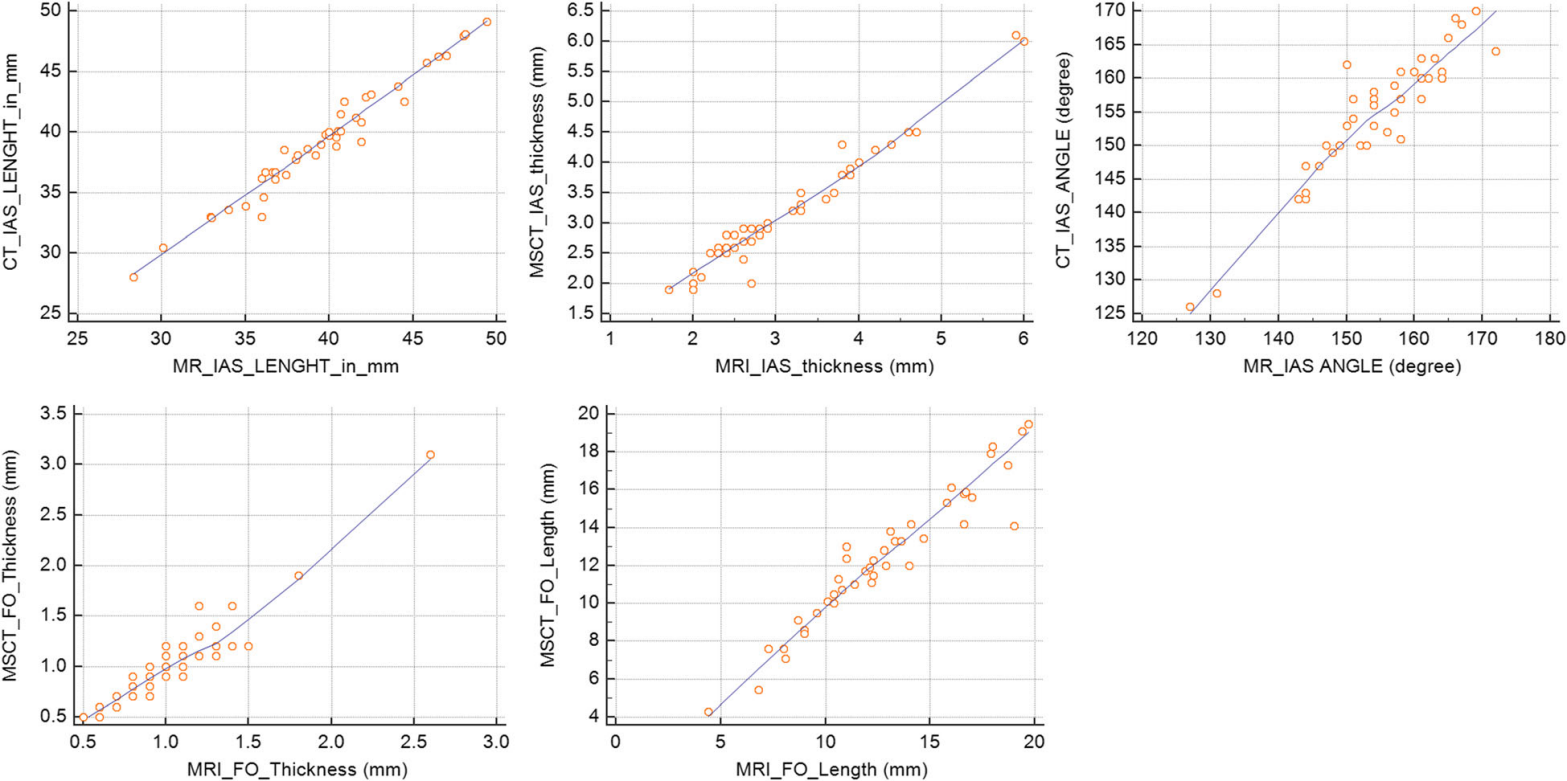
Correlation scatter graphs of both techniques. It demonstrates the close relationship between various measurements of the interatrial septum (IAS) obtained using CMR and IAS length, maximal IAS thickness, fossa ovalis thickness, fossa ovalis length, and IAS angle. FO, fossa ovalis; IAS, interatrial septum; NS, not significant

Also, we could not find a strong relation between IAS thickness and age or other patient features. Figure 3 shows the correlation graph between both groups.

## Discussion

The value of cardiac MRI compared with cardiac CT for assessment of IAS has not been addressed so far. In this study, we used special MR techniques to assess the feasibility of visualizing the measurements and morphological features of the interatrial septum by 3-T cardiac MRI technique and compare it with the data obtained by MSCT.

Variable results have been demonstrated from other studies comparing MSCT and MRI in characterization of other parts of the heart. Mahnken et al. reported excellent agreement between both techniques for characterization of left ventricular volumes and regional wall motion [16]. Similarly, Lacomis et al. found that both techniques appeared comparable for the assessment of posterior left atrial morphology [17]. On the other hand, other investigators demonstrated a discrepancy between both modalities [18, 19]. Hager et al. reported that CT and MRI are similarly useful for the non-invasive evaluation of the thoracic aorta in patients with CoA; however, there was some small variation of some measurements. They used helical CT which is an older and less accurate tool than newer MSCT scanners [18]. Also, Nonent et al. compared the concordance rates of contrast-enhanced MR angiography and CT angiography with Doppler ultrasound in carotid stenosis evaluation. They found that the concordance rate of MRI was significantly higher than that for CT in the surgical asymptomatic stenosis group [19]. Our data suggests that both techniques provide comparable information and measurements in the assessment of the interatrial septum.

### Technical considerations

The technical capabilities of cardiovascular MRI, and consequently, the clinical indications in which it can contribute to better patient management, continue to expand. Whole-heart 3-dimensional (3D) MRI at 3 T utilizing free-breathing navigator-gating has emerged in the last decade as a valuable technique in comprehensive cardiac evaluation, specially coronary artery assessment. Therefore, it would be ideal for initial and serial assessments of interatrial septum characteristics. In contrast to 1.5 T, morphological and functional cardiac imaging using various pulse sequences demonstrated noticeably improved results at 3 T when compared to 1.5 T [20, 21].

Magnetic resonance imaging at a higher field such as 3 T has the advantage of improved signal-to-noise ratio (SNR) and contrast-to-noise ratio (CNR), which can be used to enhance temporal and spatial resolution, resulting in shorter scan time and better image quality [10, 22]. Furthermore, applying parallel imaging techniques (e.g., SENSE) at 3 T has additional advantages: compensation for the decreased SNR imposed by parallel imaging and reduction of total energy deposition due to reduced scan times [23, 24]. However, cardiac imaging at 3 T exhibits some obstacles related to the static field (B0) and radiofrequency (B1) in homogeneities [25, 26] and increased sensitivity to susceptibility artifacts which may affect the image quality [27], in addition to the specific absorption rate (SAR) limitations [28].

Performing whole-heart imaging techniques is now feasible with excellent image quality [29]. Actually, enhanced spatial coverage of the thick 3D-slab can potentially improve visualization of the interatrial septum, as the two main parts of the IAS (primum and secundum septae) are not aligned within a straight line. In addition, performing whole-heart scan has the advantage of flexibility of post-processing of the acquired images. The 3D dataset can be reformatted to depict the interatrial septum. A major disadvantage of performing whole-heart imaging is the prolonged scan time compared to the volume-targeted method.

Moreover, there may be a value for use of IV-contrast material as it creates a positive blood-pool contrast, enhancing cardiac chambers in relation to the myocardium and cardiac septae. The application of an IV-contrast in combination with an IR-preparation technique for signal nulling of the myocardial wall significantly improves the contrast between the blood-pool and cardiac structures [30]. Other pulse sequences (spin echo or gradient echo) can be used successfully at 3-T MRI to visualize the anatomy and morphology of cardiac chambers and structures [20, 21]. However, when using T1-weighted images for the assessment of atrial septal defects, it has a tendency to overestimate the maximum defect diameter due to high signal dropout around the edges [31].

Recent studies have shown that SSFP images have increased signal-to-noise ratio (SNR) and increased tissue–blood contrast at 3 T compared to 1.5 T. Also, the SSFP technique provides an optimal contrast-to-noise ratio (CNR) between myocardium and blood-pool at a high SNR, allowing its practical application in the clinical setting [27, 28]. Heart–lung interface-induced B0 inhomogeneity results in black-band artifacts at high field [32]. Improving B0 homogeneity and optimization of resonance frequency could be achieved with localized linear or second-order shimming techniques which ameliorates the artifact’s effect [33]. Michaely and coworkers demonstrated the feasibility of using both SSFP and segmented spoiled gradient-echo (SGE) techniques at 3 T for cardiac function analysis with similar accuracy compared with 1.5 T [21]. In their study, 85.7% of all SSFP images at 3 T demonstrated off-resonance susceptibility artifacts at the heart–lung interface; however, they were eliminated on the repeat exams after utilizing frequency scouts to determine the optimal frequency offset. Also, they demonstrated that SGE sequences improved with higher magnetic field as a result of a 16% increase in CNR compared with 1.5 T. On the other hand, Tyler et al. advocated the use of SSFP sequences because of increased SNR and CNR than SGE sequences at 3 T, which provided higher quality images [34]. Actually, with applying better shimming techniques, using SSFP at 3 T became the most commonly used cine technique at 3 T [32].

In comparison with MSCT, CMR provided successful data acquisition without the need for ionizing radiation or potentially nephrotoxic iodinated contrast [17, 35]. Improving patient convenience is an ultimate goal in patient management, and CMR is an attractive tool for this purpose. We used the free-breathing technique, which offers an alternative high-resolution imaging technique for patients who cannot tolerate breath-holding as in MSCT [36]. Moreover, neither fasting status nor beta-blocker intake was absolutely needed in this study in preparation for CMR examination. However, these significant advantages were at the expense of longer scan time with MRI. Navigator efficiency is also an important factor that determines the whole-heart scan time; in this study, it ranges from 40 to 60% which was sufficient to minimize scanning time to an optimal value.

### Clinical implications

In the past decade, there has been a significant increase in the number of percutaneous catheter ablation procedures intervening with left atrial arrhythmias. Concurrently, several attempts were done for direct mitral valve repair with a percutaneous approach. Both procedures require an atrial transseptal puncture (ATP) to allow placing catheters in the left atrium. ATP is a procedure that necessitates traversing the interatrial septum with a needle [37], radiofrequency probe [38], or by a laser catheter [39]. This puncture needs to be confined to fossa ovalis, the thinnest part of IAS. Proper transseptal puncture could be facilitated by accurate knowledge of the anatomy and morphology of the interatrial septum. However, variations in IAS anatomy are common, including PFO [40], atrial septal aneurysm [41], and IAS lipomatous hypertrophy [42]. Identification of these differences with CMR, MSCT, or other reliable imaging methods will improve patient safety and avoid the performance of the unnecessary potentially risky procedure.

The IAS varies in thickness; the thickest parts are located peripherally at its attachment sites to the atrial free walls and gradually narrow toward the more centrally located fossa ovalis. In the current study, the IAS thickness was measured anterior and posterior to the fossa ovalis, and the mean of both values was reported. Actually, with longstanding rheumatic mitral valve disease or with previous mitral valve surgery, chronic strain forces may lead to calcification within the left atrium [43]. Harthorne et al. showed that IAS is often spared and free from calcification [44]. Increased thickness of IAS was noticed in elderly patients with atrial fibrillation (AF) using transthoracic echocardiography [45]. Xu et al. showed that changes in the atrial extracellular matrix components trigger atrial remodeling and could lead to changes in atrial wall thickness [46]. On the other hand, Galzerano et al. have demonstrated thinning of IAS eventually once sinus rhythm is restored after the conversion of AF [47].

Fender et al. found significant interpatient variation in IAS angulation [48]. Our study showed the same concept utilizing both techniques with identical measurements. For the sake of patient safety, it is a very important clinical point to know the angulation and orientation of the IAS prior to transseptal puncture, which determines optimal Brokenbrough needle curvature and angulation for the puncture.

### Limitations

We acknowledged some limitations in our study. Measurements taken by CMR were acquired with free-breathing, but MSCT were acquired with breath-hold, which might cause subtle differences in IAS measurements. Also, other techniques like transthoracic or transesophageal echocardiography could be used to assess IAS morphology. However, echocardiography does not provide tomographic imaging like CT and MRI.

The prevalence of PFO in the current study is low and does not correlate well with literature; probably, a larger sample size would be needed.

## Conclusion

Whole-heart 3D acquisition at 3-T MRI using the free-breathing technique allowed for successful evaluation of the interatrial septum, providing higher spatial resolution compared to other MRI techniques used for IAS assessment. This relatively high spatial resolution and homogenous myocardial suppression (providing high CNR) was possible due to the use of high-field MRI. These advantages allowed for a clear assessment of the IAS morphology and provided IAS measurements that are not significantly different from high-resolution CT imaging, eluding radiation exposure and iodinated contrast use. Thus, this MRI technique offers a valuable non-invasive imaging modality for excellent assessment of the interatrial septum—as compared to MSCT—which may have a significant clinical implication for diagnostic purposes and therapeutic interventional procedures, as it can facilitate planning, improve outcome, and shorten its duration.

## Data Availability

All data generated or analyzed during this study are included in this published article.

## Abbreviations

IAS: Interatrial septum
MSCT: Multi-slice computed tomography
CMR: Cardiac MRI
SNR: Signal-to-noise ratio
CNR: Contrast-to-noise ratio
3D: 3-Dimensional
VCG: Vector electrocardiographic
TD: Trigger delay
SAR: Specific absorption rate
AF: Atrial fibrillation
